# Editorial: Managing physiological and biomechanical load-adaptation pathways in high performance sport: Challenges and opportunities

**DOI:** 10.3389/fspor.2022.1041998

**Published:** 2022-10-03

**Authors:** Paul S. R. Goods, François Billaut, Franck Brocherie, Julien Louis

**Affiliations:** ^1^Murdoch Applied Sports Science Laboratory, Murdoch University, Perth, WA, Australia; ^2^Centre for Healthy Ageing, Health Futures Institute, Murdoch University, Perth, WA, Australia; ^3^Department of Kinesiology, Laval University, Quebec City, QC, Canada; ^4^Laboratory Sport, Expertise and Performance (EA 7370), French Institute of Sport (INSEP), Paris, France; ^5^Research Institute for Sport and Exercise Sciences, Liverpool John Moores University, Liverpool, United Kingdom

**Keywords:** training load, external load, training monitoring, training adaptation, ergogenic aids

## Background

High performance sport is continuing to push the barriers of elite athletes' physiological and biomechanical adaptation in an effort to gain an edge in highly competitive sporting environments, where the winning margins can be extremely thin. Methods for individualizing and optimizing the load-adaptation relationship to different training interventions have long been sought (1, 2), and continue to this day (3, 4). But while physiological adaptations to training have been well-studied, differentiating between physiological and biomechanical load-adaptation pathways is vital for understanding how best to optimize individualized training programs (5). Also of significant interest is the use of ergogenic aids such as dietary supplements (6), environmental manipulation (7), or sophisticated footwear designs (8) on physiological and biomechanical training adaptations, respectively. In addition to understanding load-adaptation pathways, and of equal importance, is determining rigorous methodology for assessing training load, so that the load-adaptation relationship can be better quantified (9, 10). However, despite the growing interest in training and ergogenic intervention research, there remains much to be learnt about assessing and managing the load-adaptation pathway within elite athlete populations.

## Featured publications

This Research Topic of articles attempts to resolve current challenges and opportunities in understanding physiological and biomechanical load-adaptation pathways. The article by Ruf et al. entitled “*Are measurement instruments responsive to assess acute responses to load in high-level youth soccer players?*” analyzed the short-term responsiveness of measurement instruments for quantifying psycho-physiological responses to training load. The authors found that athlete-reported ratings of stress and recovery, countermovement jump parameters, exercising heart rate and heart rate recovery demonstrated only small-to-moderate relationships with the accumulation of training load. Importantly, training load indicators such as total distance, high-speed running distance (>4.7 m.s^−1^), as well as the product of session rating of perceived exertion (sRPE) and session duration (in minutes) were unable to provide an accurate estimate of the mechanical demands of training. The results of this investigation bring into question the utility of these common measurement instruments for quantifying training-induced stress, and the authors therefore caution against making training load adjustments based on such values only.

Miguel et al., in their article entitled “*Daily and weekly external loads in the microcycle: Characterization and comparison between playing positions on amateur soccer*,” attempted to overcome some of the methodological challenges in designing and quantifying training programs for soccer players. To do this, training load was broken down into sprint distance, moderate- and high-intensity accelerations and decelerations, with data displayed relative to match-day reference values. This approach allowed the authors to describe a training load model that contemplates the individualization of the physical demands of the match, for each playing position, and for every individual.

Similarly,  Byrkjedal et al., in their article entitled “*Simulated game-based ice hockey match design (scrimmage) elicits greater intensity in external load parameters compared with official matches*,” delineated training load into several intensity bands based on velocity, acceleration and inertial measurement unit variables to determine whether simulated match performances could replicate the training load demands of official matches in ice hockey. Despite similar match durations and playing times, the authors reported that simulated matches were able to elicit greater high-intensity training loads than official matches, with 10.0–24.1% increases in high-intensity events, decelerations, changes of direction and high-speed skating. Overall, these findings highlight the need to utilize more informative metrics such as acceleration load, changes of direction, and sprints when describing team-sport training loads.

Finally,  Cyr-Kirk and Billaut, in their article entitled “*Hyperoxia improves repeated-sprint ability and the associated training load in athletes*,” investigated the impact of environmental manipulation on the acute training load in team- and racquet-sport athletes. Supplementation with a hyperoxic gas mixture (F_I_O_2_ = 0.40) during exhaustive repeated-sprint exercise increased the total work performed during the set and the associated training load (assessed *via* multiplying total mechanical work by sRPE). The authors argued that performing more repetitions during repeated-sprint exercise could trigger mechanical and/or physiological changes that, over time, should enhance the neuromuscular function and exercise capacity. However, whether such a training approach could lead to superior adaptations requires a more robust assessment in periodized, long-term investigations.

Collectively, the findings presented in this Research Topic highlight the need for practitioners to utilize valid and reliable training load metrics which are sensitive to changes in training load for a given sporting context. The use of multiple intensity bands for acceleration- and velocity-based metrics reported in team sport investigations are examples of this in the current Research Topic. Additionally, it is paramount that physiological, psycho-physiological and biomechanical training load indices be based on a scientific understanding of the specific demands of the sport. This will require researchers and practitioners to first investigate these sport-specific demands before the load-adaptation relationship to any intervention can be examined. When these fundamental principles of applied sport science are adhered to, practitioners are able to better determine the impacts of various training or ergogenic interventions on subsequent training load, and ultimately physiological and biomechanical adaptations and resultant performance.

## Author contributions

All authors listed have made a substantial, direct, and intellectual contribution to the work and approved it for publication.

## Conflict of interest

The authors declare that the research was conducted in the absence of any commercial or financial relationships that could be construed as a potential conflict of interest.

## Publisher's note

All claims expressed in this article are solely those of the authors and do not necessarily represent those of their affiliated organizations, or those of the publisher, the editors and the reviewers. Any product that may be evaluated in this article, or claim that may be made by its manufacturer, is not guaranteed or endorsed by the publisher.
